# Facile Synthesis of Battery-Type CuMn_2_O_4_ Nanosheet Arrays on Ni Foam as an Efficient Binder-Free Electrode Material for High-Rate Supercapacitors

**DOI:** 10.3390/nano13061125

**Published:** 2023-03-21

**Authors:** Chandu V. V. Muralee Gopi, R. Ramesh, Rajangam Vinodh, Salem Alzahmi, Ihab M. Obaidat

**Affiliations:** 1 Department of Electrical Engineering, University of Sharjah, Sharjah P.O. Box 27272, United Arab Emirates; 2 Department of Chemical Engineering, School of Mechanical, Chemical and Materials Engineering, Adama Science and Technology University, Adama P.O. Box 1888, Ethiopia; 3 Green Hydrogen Lab (GH2Lab), Institute for Hydrogen Research (IHR), Université du Québec à Trois-Rivières (UQTR), 3351 Boulevard des Forges, Trois-Rivières, QC G9A 5H7, Canada; 4 Department of Chemical & Petroleum Engineering, United Arab Emirates University, Al Ain P.O. Box 15551, United Arab Emirates; 5 National Water and Energy Center, United Arab Emirates University, Al Ain P.O. Box 15551, United Arab Emirates; 6 Department of Physics, United Arab Emirates University, Al Ain P.O. Box 15551, United Arab Emirates

**Keywords:** CuMn_2_O_4_, nanosheet arrays, hydrothermal, battery-type, supercapacitors

## Abstract

The development of battery-type electrode materials with hierarchical nanostructures has recently gained considerable attention in high-rate hybrid supercapacitors. For the first time, in the present study novel hierarchical CuMn_2_O_4_ nanosheet arrays (NSAs) nanostructures are developed using a one-step hydrothermal route on a nickel foam substrate and utilized as an enhanced battery-type electrode material for supercapacitors without the need of binders or conducting polymer additives. X-ray diffraction, scanning electron microscopy (SEM), and transmission electron microscopy (TEM) techniques are used to study the phase, structural, and morphological characteristics of the CuMn_2_O_4_ electrode. SEM and TEM studies show that CuMn_2_O_4_ exhibits a nanosheet array morphology. According to the electrochemical data, CuMn_2_O_4_ NSAs give a Faradic battery-type redox activity that differs from the behavior of carbon-related materials (such as activated carbon, reduced graphene oxide, graphene, etc.). The battery-type CuMn_2_O_4_ NSAs electrode showed an excellent specific capacity of 125.56 mA h g^−1^ at 1 A g^−1^ with a remarkable rate capability of 84.1%, superb cycling stability of 92.15% over 5000 cycles, good mechanical stability and flexibility, and low internal resistance at the interface of electrode and electrolyte. Due to their excellent electrochemical properties, high-performance CuMn_2_O_4_ NSAs-like structures are prospective battery-type electrodes for high-rate supercapacitors.

## 1. Introduction

The exhaustion of fossil fuels, with related global climate issues and growing energy demand from society, has created an urgent call for the progress of high-rate electrochemical energy storage systems that can store vast amounts of energy (high specific energy) and have rapid charge–discharge (high specific power) [1]. Currently, lithium-ion batteries (LIB), supercapacitors (SCs), and sodium-ion (SIBs) storage systems are extensively used for the storage systems of electric vehicles, portable electronics, wearable electronics, etc. [2,3]. Supercapacitors have received the most attention from people among all types of energy storage devices, owing to their benefits including outstanding cycling stability, superior specific power, and rapid charge–discharge cycles [4]. Supercapacitors are classified into three varieties based on their energy storage capabilities: electrochemical double-layer capacitors (EDLCs), pseudocapacitors (PCs), and battery-type supercapacitors [5]. The EDLC materials accumulate the charges by the adsorption of electrolyte ions at the electrode and electrolyte interface, while the PCs and battery-type materials store charges by Faradaic electrochemical reactions. The morphology, structure, and conductivity of the electrode materials, the electrolyte, the complexity of the device’s fabrication circumstances, and other aspects need to be considered while preparing supercapacitor devices. The electroactive material is the essential supercapacitor device component to determine electrochemical performance [6]. Commonly used electrode materials include conductive polymers, conductive carbon compounds, transition metal components, etc. [7]. However, the SCs could produce a tremendous specific power but low specific energy; in contrast, the LIBs could only give a low specific power [8,9]. In this case, creating a hybrid supercapacitor (HSC) with high specific power and high specific energy is particularly desirable [10]. These HSCs comprise a collective arrangement of a supercapacitor electrode material and a battery-type electrode material [11]. Hence, in recent years developing positive and negative electroactive materials for HSC has witnessed significant demand in electric vehicles, portable electronics, and wearable electronics applications.

Battery-type materials, such as NiO, Co_3_O_4_, Ni(OH)_2_, NiS, NiCo_2_S_4_, etc., delivered superior energy storage capabilities compared to electric double layer capacitors (EDLC materials, such as carbon-related materials, such as reduced graphene oxide, graphene, etc.) and pseudocapacitor materials (such as MnO_2_, RuO_2_, etc.) [12,13,14,15,16,17]. Therefore, it is crucial to design and create highly effective battery-type electrodes to elevate the supercapacitor’s performance, particularly its specific energy. Additionally, binary metal oxides (CuCo_2_O_4_, NiCo_2_O_4_, NiMoO_4_, CoMoO_4_, FeMoO_4_, CuNiO_2_, NiMn_2_O_4_, MnCo_2_O_4_, etc.) have drawn significant interest as battery-type electrodes for supercapacitors compared to the mono-metal oxides due to their enhanced electrical conductivity and additional oxidation states [18,19,20,21]. Manganese-based binary oxides have received the most attention among these oxides, because they offer numerous benefits including low toxicity, great abundance, various valence, and low cost [22]. Several research initiatives have been developed to produce binary metal oxides, including exploring novel electrode materials and constructing hierarchical nanostructures. For example, Krishnan et al. developed a MnCo_2_O_4_ nanoflakes battery-type material for a supercapacitor using the rapid microwave-assisted technique and obtained a specific capacity of 74.44 mA h g^−1^ at 0.5 A g^−1^ [23]. The NiMn_2_O_4_ synthesized by Krishna et al. delivered a specific capacity of 33.66 mA h g^−1^ at 0.5 A g^−1^ [24]. By a simple hydrothermal route, Wei et al. developed the nanostructured spinal NiMn_2_O_4_ electrode and obtained a specific capacity of 73.61 mA h g^−1^ at 1 A g^−1^ with superb cycling stability (96% over 1000 cycles) [25]. Recently, Cheng et al. synthesized hierarchical CuMn_2_O_4_ microspheres using a micro/nano MnCO_3_ precursor and obtained a specific capacity of 73 mA h g^−1^ at 1 A g^−1^ [26]. On the other hand, Zhang et al. deposited the spinel CuMn_2_O_4_ on graphene nanosheets (CuMn_2_O_4_-RGO) using the sol-gel method and physical grinding and obtained a specific capacity of 95 mA h g^−1^ at 1 A g^−1^ [27]. However, it still remains a challenge to develop CuMn_2_O_4_ micro/nanostructures with outstanding energy storage performance for supercapacitors. Moreover, transition metal oxide performance is unsatisfactory in supercapacitor applications due to their limited electrical conductivity, intrinsic structural features, and lower capacitance value than the theoretical value. Therefore, exceptional efforts have been undertaken in this direction to create battery-type binary metal oxides with various morphologies for high-rate supercapacitors.

It is also necessary to consider the fabrication route to develop highly efficient electrode materials for hybrid supercapacitor applications. Several fabrication routes for producing metal oxides and sulfides include sol-gel, coprecipitation, chemical bath deposition, and hydrothermal and solvothermal reactions. Among these approaches, hydrothermal synthesis typically outperforms the others because of its low-temperature synthesis process, affordable equipment, and ability to modify composition and particle size by adjusting fabrication factors [16,28]. As a result, a simple hydrothermal approach was applied in this study to create a highly effective electrode material for supercapacitor applications.

Inspired by the latest findings of binary metal oxides, we created a CuMn_2_O_4_ nanosheet-like structure using a facile one-step hydrothermal approach and effectively utilized it as a battery-type electrode for supercapacitors. According to structural and morphological investigations, CuMn_2_O_4_ has a nanosheet array-like morphology that provides plenty of electroactive sites and promotes quick redox reactions. As a result, the battery-type CuMn_2_O_4_ NSA electrode displays an exceptional specific capacity (125.56 mA h g^−1^ at 1 A g^−1^), excellent rate capability (84.1% even at 10 A g^−1^), and extraordinary cycling performance (92.15% after 5000 cycles). Moreover, at various bending angles (flat, 45°, and 90°) the CV and GCD plots of the CuMn_2_O_4_ NSAs electrode are consistent in shape and no noticeable distortion occurs, suggesting outstanding mechanical stability and flexibility.

## 2. Experimental Method

### 2.1. Materials

The chemicals used in this study were acquired from Sigma-Aldrich, Seoul, South Korea, and utilized without additional purification; these are potassium hydroxide (KOH), copper nitrate hexahydrate (Cu(NO_3_)_2_·6H_2_O), ammonium fluoride (NH_4_F), manganese nitrate hexahydrate (Mn(NO_3_)_2_·6H_2_O), hydrochloric acid (HCl), and urea (CH_4_N_2_O).

### 2.2. Fabrication of Battery-Type CuMn_2_O_4_ NSA Material on Ni Foam Surface

On nickel (Ni) foam substrate, binder-free CuMn_2_O_4_ NSA structures were deposited via a simple one-step hydrothermal technique. The 1 × 2 cm^2^ Ni foam substrates were cleaned using an ultrasonic cleaner for 15 min each with 3 M HCl, acetone, ethanol, and deionized (DI) water before the electroactive material was deposited. Based on our previous work, various binary metal oxides were deposited on the Ni foam surface at the deposition temperature and time of 100 °C and 6 h. Hence, in the present study the deposition temperature and time of 100 °C and 6 h are used in the hydrothermal condition to deposit the CuMn_2_O_4_ NSA material on the Ni foam surface [20,29,30]. In 60 mL DI water, 0.05 M of Cu(NO_3_)_2_·6H_2_O, 0.1 M of Mn(NO_3_)_2_·6H_2_O, 0.24 M of CH_4_N_2_O and 0.12 M NH_4_F were combined and stirred for 30 min to deposit the CuMn_2_O_4_ material on Ni foam. Pre-cleaned Ni foams and the CuMn_2_O_4_ reaction recipe were put into a 100 mL Teflon-lined autoclave for 6 h at 100 °C. After cooling, the electrodes were taken out of the autoclave, washed several times with deionized water and ethanol, and dried at 60 °C for a whole night. Ultimately, the electrode was heated for 2 h at 200 °C and given the designation CuMn_2_O_4_ NSAs. The active material loading on Ni foam is about 2.3 mg. The CuO material on Ni foam was also synthesized at the same fabrication method, except without the addition of Mn(NO_3_)_2_·6H_2_O [31].

### 2.3. Material Characterization and Electrochemical Measurements

Transmission electron microscopy (TEM, CJ111), X-ray diffraction (D8 ADVANCE), and scanning electron microscopy (SEM, S4800, Hitachi, Pusan National University, Busan, South Korea) were used to analyze the morphology, crystalline structure, and phase purity of the as-prepared CuMn_2_O_4_ electrode. All electrochemical measurements were made using a Bio-Logic SP-150 electrochemical analyzer. In a three-electrode system configuration filled with 3 M KOH aqueous electrolyte, the electrochemical behaviors of the as-developed electrode were examined utilizing electrochemical impedance spectroscopy (EIS), galvanostatic charge–discharge (GCD), and cyclic voltammetry (CV). The as-prepared CuMn_2_O_4_ electrode was used as the working electrode, Ag/AgCl electrode was used as the reference electrode, and the platinum wire was used as the counter electrode. In a three-electrode arrangement, Equation (1) was used to compute the specific capacity (Q_SC_, mA h g^−1^) of the as-fabricated sample from the GCD plots [32].

(1) $$Q_{SC}=\frac{I\times \Delta t}{m\times 3.6}$$

where *I* (A) is the current (A), Δ*t* (s) is the discharge time, and *m* (g) is the electroactive material mass (g).

## 3. Results and Discussion

**Figure 1:**
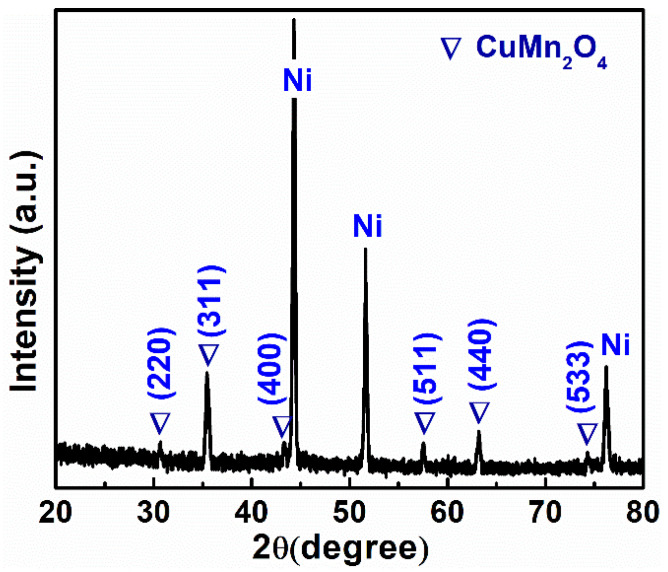
CuMn_2_O_4_ NSA’s XRD pattern on the surface of Ni foam.

To validate the effective deposition of the CuMn_2_O_4_ active material on the Ni foam, an XRD analysis of the thin film was performed. Figure 1 shows the XRD spectrum of CuMn_2_O_4_, which displayed well-defined sharp peaks consistent with the previous literature reports [33,34], denoting the successful fabrication of the active material. Bright and intense Nickel diffraction peaks are visible in XRD due to the backdrop of Ni foam. The peaks obtained at 2θ positions of 30.6°, 35.6°, 43.4°, 57.5°, 63.2°, and 74.3° correspond to the (220), (311), (400), (511), (440), and (533) planes of crystalline CuMn_2_O_4_ (JCPDS no. 34-1400) [33,34]. The absence of additional peaks in the XRD pattern supports the CuMn_2_O_4_ sample’s phase purity.

**Figure 2:**
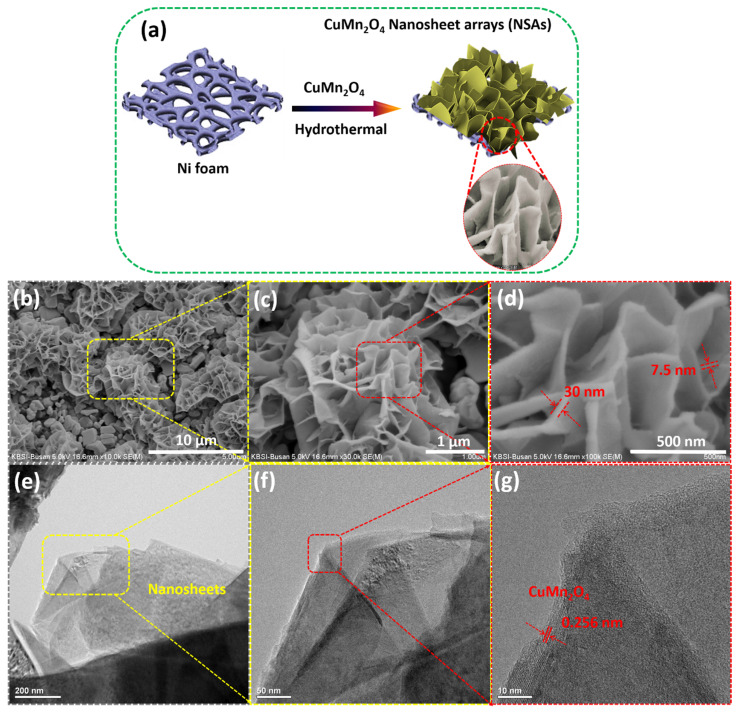
(**a**) Schematic diagram of the preparation of CuMn_2_O_4_ NSAs electrode. (**b**–**d**) SEM and high-resolution SEM images of CuMn_2_O_4_ NSAs electrode. (**e**–**g**) TEM and high-resolution TEM images of CuMn_2_O_4_ NSAs electrode.

Figure 2a depicts the schematic representation for the fabrication of binder-free CuMn_2_O_4_ nanosheet arrays on the Ni foam using a simple one-step hydrothermal. Ni foam, as it is widely known, is a three-dimensional (3D) structure that is highly conductive and possesses an open porous structure with huge surface area stems. As a result, Ni foam could be utilized as an efficient conductive stage to increase the active material mass loading, and is also more suited for the sweep out of electrons produced from the electroactive material during redox processes. These fantastic benefits led us to select the Ni foam as a current collector and build the CuMn_2_O_4_ NSA sequentially using a one-step hydrothermal method. Urea and NH_4_F were utilized as precipitants and complexing agents, respectively. 

As shown in Figure 2b–d, the morphology and structure of the fabricated CuMn_2_O_4_ electrode were examined by SEM analysis. The SEM image with low magnification (Figure 2b) shows that the CuMn_2_O_4_ active material is completely coated on the Ni foam surface. The high-resolution SEM images in Figure 2c,d reveal that the microstructures are made up of interwoven ultrathin nanosheets with relative thicknesses ranging from ~7.5 to 30 nm. The ultrathin nanosheets are interconnected and generate a hierarchical nanosheet array (NSA) morphology. TEM and high-resolution-TEM characterization methods were used to evaluate the morphology and crystalline characteristics of the CuMn_2_O_4_ NSAs electrodes. Different magnification TEM pictures of CuMn_2_O_4_ NSAs are shown in Figure 2e–g, and they demonstrate the deposition of hierarchical CuMn_2_O_4_ NSAs over the Ni foam surface. The HR-TEM picture exhibits lattice fringes of the CuMn_2_O_4_ compound at a lattice spacing of 0.256 nm relating to the (311) plane, as shown in Figure 2g. The TEM results are consistent with the XRD analysis of the CuMn_2_O_4_ NSAs. The as-deposited NSAs are anticipated to improve the specific surface area, supply many electroactive sites, and enable the rapid diffusion of electrolyte ions, all of which will enhance the charge storage performance.

**Figure 3:**
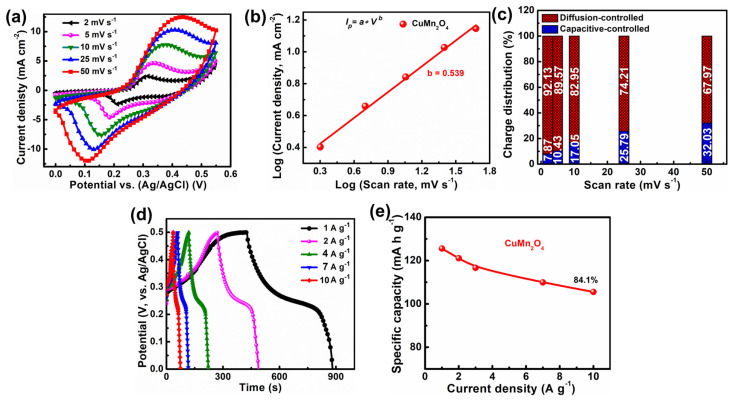
(**a**) CuMn_2_O_4_ NSAs electrode CV graphs at different sweep rates. (**b**) The b−value was calculated using a Log of cathodic peak current density vs. a Log of sweep rate. (**c**) The plot of the CuMn_2_O_4_ NSAs electrode’s charge distribution vs. scan rates. (**d**) CuMn_2_O_4_ NSAs electrode GCD profiles at varied current densities. (**e**) Calculated specific capacity values vs. current density of the CuMn_2_O_4_ NSAs electrode.

The electrochemical battery-type supercapacitor performance of the CuMn_2_O_4_ NSAs electrode was investigated by CV, GCD, and EIS techniques in a three-electrode configuration using 3 M KOH as the electrolyte. As-prepared CuMn_2_O_4_ NSAs electrodes belong to the battery-type electrodes, as shown by the CV plots of the CuMn_2_O_4_ NSAs sample in Figure 3a, which show clear redox peaks obtained in the potential window range from 0 to 0.55 V (vs. Ag/AgCl) at various sweep rates of 2, 5, 10, 25, and 50 mV s^−1^. The diffusion mechanism is mainly due to the redox reaction between the electrolyte ions and the active material. As a result, diffusion control mainly drives the active substance’s contribution to specific capacity at low scan rates. Peak current levels of the pair of redox peaks were seen to increase as the scan rate increased, and the forms of the redox peaks were conserved even at a high sweep rate of 50 mV s^−1^, implying a rapid redox reaction, elevated electro-conductivity, and excellent rate capacity of this electrode. Additionally, the oxidation and reduction peaks gradually moved to more positive and negative potentials when the sweep rate rose because of the electrochemical redox reaction’s fast charge and discharge rates and lower material resistance [35]. According to the following equations, the reversible Faradaic redox reactions of Cu and Mn species provide the basis for the electrochemical reaction’s mechanism:

CuMn_2_O_4_ + OH^−^ + H_2_O ⇋ CuOOH + 2MnOOH + e^−^ (2)

CuOOH + OH^−^ ⇋ CuO_2_ + H_2_O + e^−^ (3)

MnOOH + OH^−^ ⇋ MnO_2_ + H_2_O + e^−^ (4)

Additionally, to determine the charge storage kinetics of the as-developed material, the *b* value was calculated using log (*i*) = blog(*v*) + log(*a*) where *v and I* denote the cathode sweep rate and peak current, respectively [36]. As depicted in Figure 3b, the *b* value of the CuMn_2_O_4_ NSAs electrode material is 0.539, which is very close to 0.5, indicating that its diffusion-control (battery-type) dominant kinetics behavior is entirely compatible with recent studies on battery-type material behavior [18,36].

Furthermore, to estimate the diffusive- and capacitive-controlled contribution in the CuMn_2_O_4_ NSAs electrode, we represented peak current (*i_p_*) as the sum of a capacitive-regulated (*k*_1_*v*) and diffusion-regulated (*k*_2_*v*) process by the following Equations [37]:

*i*_p_ = *k*_1_*v* + *k*_2_*v*^1/2^ (5)

(6) $$\frac{i_{p}}{v^{1/2}}=k_{1}v^{1/2}+k_{2}\;$$

where *i_p_*, υ, and *k*_1_ and *k*_2_ represent the peak current (A), sweep rate (V s^−1^), and the constant parameters. The linearly fitted plot between *i/v*^1/2^ vs. *v*^1/2^ provided the *k*_1_ and *k*_2_ values. As illustrated in Figure 3c, the CuMn_2_O_4_ NSAs electrodes demonstrated a more dominating diffusion-controlled mechanism, with 92.13% overall capability at a low sweep rate of 2 mV s^−1^. The intense battery-type mechanism was caused by the electrolyte’s OH- ions having sufficient time to diffuse into the electrode material at low sweep rates. However, the CuMn_2_O_4_ NSAs electrode’s diffusion-controlled contribution was reduced to 67.97% as the sweep rate rose from 2 to 50 mV s^−1^, while the capacitive-controlled impact climbed to 32.03%. With increased scan rates, the reduced diffusion-controlled behavior was due to insufficient time for ion migration and intercalation; in contrast, the enhanced capacitive-regulated behavior was due to the rapid electrolyte ions transit that happens at the interface of electrode and electrolyte.

The GCD plots of the CuMn_2_O_4_ NSAs electrode are shown in Figure 3d, which depicts the current densities varying from 1 to 10 A g^−1^ with a potential window range of 0 to 0.5 V. The GCD plots’ distinct plateau sections reveal their battery-like nature. The results of the GCD tests on the CuMn_2_O_4_ NSAs electrode agree well with those obtained from the CV. Due to the limited ion diffusion at high current density, the charging and discharging periods for the CuMn_2_O_4_ NSAs electrode steadily reduce as the current density increases. However, the ions and charges in the electrolyte will have enough time to diffuse and transfer when the current density is low [38]. Equation (1) was used to determine the specific capacities of the battery-type CuMn_2_O_4_ NSAs electrode at different current densities, and calculated values are depicted in Figure 3e. The calculated specific capacities of the CuMn_2_O_4_ NSAs electrode were 125.56, 121.11, 116.67, 109.92, and 105.56 mA h g^−1^ at 1, 2, 4, 7, and 10 A g^−1^, respectively. As investigated in the electrochemical analyses, the CuMn_2_O_4_ NSA electrode delivered a high Q_SC_ value of 125.56 mA h g^−1^ at a current density of 1 A g^−1^. Furthermore, the CuMn_2_O_4_ NSAs electrode material retained 84.1% of its initial specific capacity value even at 10 A g^−1^ current density, indicating the nanostructured CuMn_2_O_4_ electrode materials’ high charge–discharge efficiency. The specific capacity value’s steady reduction with increasing current density was mainly due to the loss of active materials on the Ni foam surface during the redox reaction [39]. The specific capacity values produced in this study are comparable to and even more significant than the other electroactive materials and other reported CuMn_2_O_4_ hierarchical structures for supercapacitors previously reported (Table 1). 

**Figure 4:**
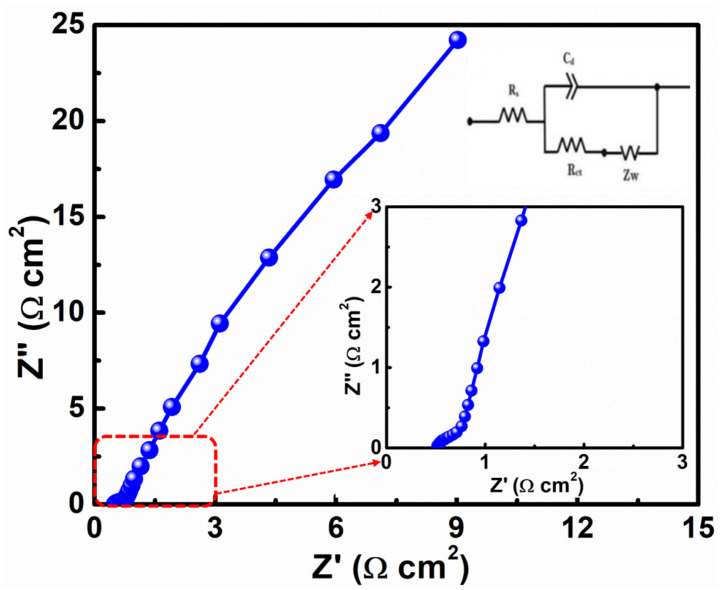
Nyquist plot of CuMn_2_O_4_ NSAs electrode (inset shows the enlarged Nyquist plot and equivalent circuit to fit the Nyquist plots).

Moreover, the CuMn_2_O_4_ NSAs electrode was further investigated using EIS analysis to assess the material’s charge transfer kinetics and electrical conductivity. Figure 4 depicts the resulting Nyquist plot of the as-fabricated CuMn_2_O_4_ NSA electrode in the 0.01 Hz–100 kHz frequency range at the open-circuit potential. The obtained Nyquist plots are fit using the equivalent circuit shown in the inset of Figure 4. The Nyquist plot showed a small semi-circle in the high-frequency and a straight line in the low-frequency regions, representing the charge transfer resistance (R_ct_) and Warburg diffusion resistance, respectively. The intersection of the Nyquist plot with the X-axis in the high-frequency area represents the electrode material’s equivalent series resistance (R_s_). The fitting results show that low R_S_ (∼0.58 Ω cm^2^) and R_ct_ (∼0.18 Ω cm^2^) values on the CuMn_2_O_4_ NSAs electrode demonstrated excellent charge transfer kinetics and outstanding electronic conductivity. The vertical line in the low-frequency zone represents the lower Warburg diffusion resistance, representing the quick electrolyte diffusion and rapid ion charge transfer. The slope for the CuMn_2_O_4_ NSA electrode is near 45°, showing that the Faradic redox reaction for the CuMn_2_O_4_ NSA is mainly controlled by diffusion, indicating that the CuMn_2_O_4_ NSA corresponds to conventional battery-type electrode materials, as depicted in Figure 4.

**Figure 5:**
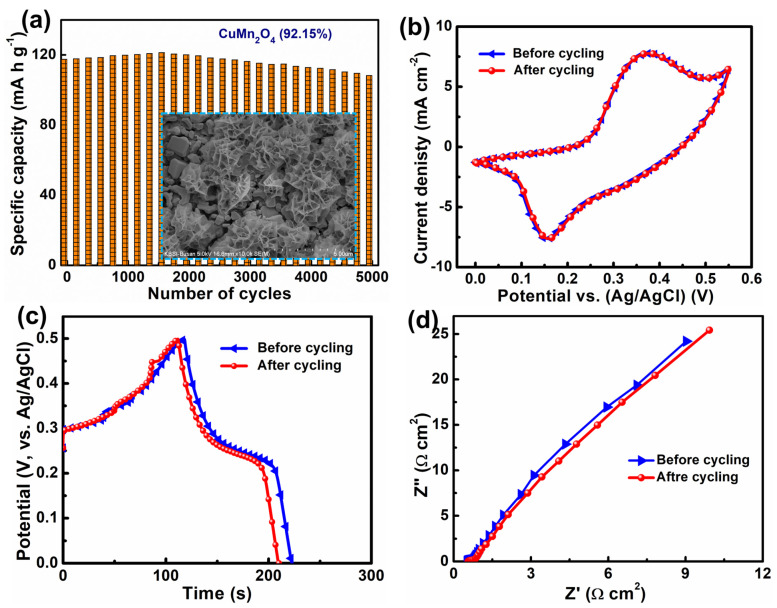
(**a**) Cycling stability (inset depicts the SEM image after the stability test). The CuMn_2_O_4_ NSA electrode (**b**) CV, (**c**) GCD, and (**d**) EIS graphs before and after cycle testing.

Furthermore, the electroactive material’s excellent cycling behavior is essential for supercapacitors’ real-time application. The cycling behavior of the CuMn_2_O_4_ NSAs material evaluated at 4 A g^−1^ over 5000 cycles is shown in Figure 5a. The specific capacity values improved somewhat in the early cycles due to the complete activation of the CuMn_2_O_4_ NSAs material by the continual entry of electrolyte ions into their inner portions [40]. Over 5000 cycles, the specific capacity gradually decreased and it retained ∼92.15% of its primary specific capacity value, demonstrating exceptional cycling life. This was further verified by the SEM picture of the CuMn_2_O_4_ electrode taken after 5000 cycles (inset of Figure 5a). The shape of the nanosheet arrays was highly retained, and the electroactive material remained attached to the surface of the Ni foam, illustrating the remarkable cycling life of the CuMn_2_O_4_ NSAs electrode. Moreover, electrochemical measurements (CV, GCD, and EIS) were carried out both before and after the cycling assessment, and the related graphs are shown in Figure 5b–d. As shown in Figure 5b,c, before and after the cycling test, the CV and GCD curves preserved no change in the energy storage performance, revealing the excellent cycling stability of the electroactive material on the Ni foam surface. As shown in Figure 5d, the obtained R_S_ values before and after the cycling test were almost the same, denoting the stable electrochemical stability of the electroactive material. Moreover, EIS analysis reveals a minor change in the charge transfer resistance of the CuMn_2_O_4_ NSA electrode after the cycling test (∼0.18 Ω cm^2^ to ∼0.29 cm^2^). Hence, the slightly decreased specific capacity value and a slight rise in R_ct_ value during the cycling test might be attributed to a slight solidification or dryness of the electrolyte [41]. Moreover, the tangent line in the low-frequency region of the Nyquist plot confirms the lower Warburg diffusion resistance, which facilitates the ionic diffusion process. 

The CuMn_2_O_4_ NSAs electrode delivered an excellent specific capacity, outstanding rate capability (84.1%), and good cycling life span. It is worth mentioning that the delivered higher *specific capacity* value from the hydrothermally fabricated battery-type CuMn_2_O_4_ NSA electrode exhibited improved electrochemical performance that was analogous to the performances of the previously reported battery-type binary metal oxide electrodes such as the hydrothermally prepared NiCo_2_O_4_ electrode (Q_SC_ = 34.02 mA h g^−1^ at 1 A g^−1^ and cycling life of 78.30% over 6000 cycles) [42], hydrothermally developed CuCo_2_O_4_ electrode (Q_SC_ = 84.22 mA h g^−1^ at 1 A g^−1^ and cycling life of 71.80% over 5000 cycles) [43], microwave-assisted NiMn_2_O_4_ electrode (Q_SC_ = 138.83 mA h g^−1^ at 1 A g^−1^ and cycling life of 85.80% over 6000 cycles) [44], hydrothermally synthesized CuNiO_2_ electrode (Q_SC_ = 111.52 mA h g^−1^ at 2 A g^−1^ and cycling life of 89.13% over 3000 cycles) [45], hydrothermally prepared CuCo_2_O_4_ electrode (Q_SC_ = 86.37 mA h g^−1^ at 1 A g^−1^ and cycling life 93% over 6000 cycles) [46], and the hydrothermally developed FeCo_2_O_4_ electrode (Q_SC_ = 125.56 mA h g^−1^ at 1 A g^−1^ and cycling life 93.68% over 4000 cycles) [47], respectively. Table 1 compares and summarizes the total electrochemical behavior of different electrodes.

**Table 1:** The energy storage behavior of the current battery-type CuMn_2_O_4_ NSAs electrode and previously disclosed battery-type binary metal oxide electrodes and other reported CuMn_2_O_4_ hierarchical structures are compared.

Battery-Type Electrode	Preparation Route	Specific Capacity (mA h g^−1^)	Cycling Stability (Cycles)	Ref.
NiCo_2_O_4_ flower-like	Hydrothermal	34.02 at 1 A g^−1^	78.30% (6000)	[42]
CuCo_2_O_4_ ultrathin nanosheets	Hydrothermal	84.22 at 1 A g^−1^	71.80% (5000)	[43]
NiMn_2_O_4_ microspheres	Microwave-assisted	138.83 at 1 A g^−1^	85.80% (6000)	[44]
CuNiO_2_ dandelion flower-like	Hydrothermal	111.52 at 2 A g^−1^	89.13% (3000)	[45]
CuCo_2_O_4_ microspheres	Hydrothermal	86.37 at 1 A g^−1^	93.00% (6000)	[46]
FeCo_2_O_4_ chopsticks-like	Hydrothermal	113.32 at 1 A g^−1^	93.68% (4000)	[47]
CuMn_2_O_4_ microspheres	Micro/nano MnCO_3_ precursor	73 at 1 A g^−1^	96% (3000)	[26]
Spinel CuMn_2_O_4_-RGO nanosheets	Sol-gel with physical griding	95 at 1 A g^−1^	75.5% (1000)	[27]
CuMn_2_O_4_	Hydrothermal	125.56 at 1 A g^−1^	92.15% (5000)	Present work

**Figure 6:**
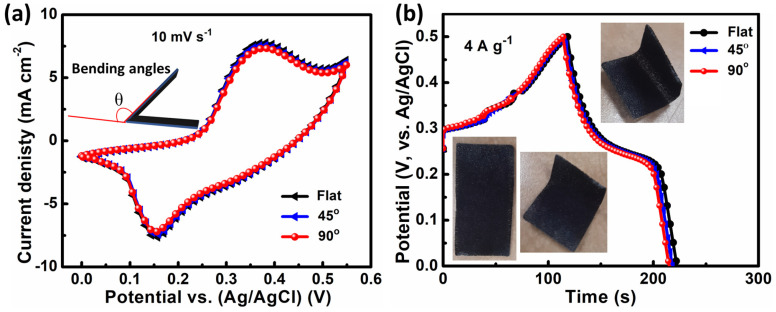
At different bending angles, the (**a**) CV and (**b**) GCD profiles of a CuMn_2_O_4_ NSA electrode. Photographs of CuMn_2_O_4_ NSA electrode bent in various angles showing the flexibility of the electrode (inset of Figure 6b).

Moreover, CV and GCD assessments at different bending angles were used to examine the CuMn_2_O_4_ NSA electrode’s flexibility and mechanical stability. The obtained curves are depicted in Figure 6a,b. The inset of Figure 6b shows the photographs of the CuMn_2_O_4_ NSA electrode bent at various angles. The CuMn_2_O_4_ NSA electrode is highly flexible and can be bent at 45° and 90° angles without destroying its physical structure, as is shown in the inset of Figure 6b. The CuMn_2_O_4_ electrode’s CV and GCD profiles exhibit almost identical CV and GCD profiles at different bending angles (flat, 45°, and 90°), and show minimal performance change, demonstrating the material’s exceptional mechanical stability and flexibility. 

**Figure 7:**
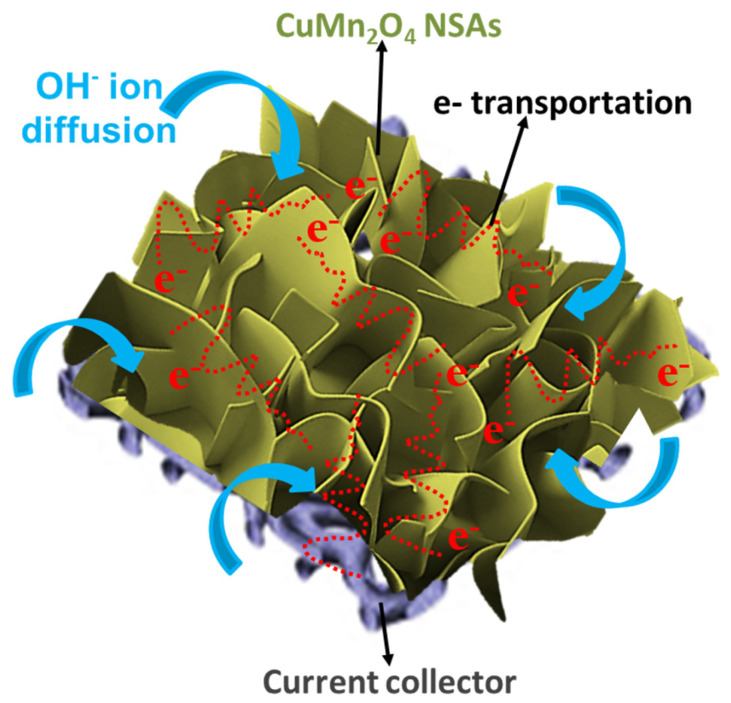
Diffusion of electrolyte ions is shown schematically in CuMn_2_O_4_ nanosheet array morphology.

Figure 7 depicts the electrochemical behavior of the binder-free CuMn_2_O_4_ NSAs material on Ni-foam for the hybrid supercapacitors. The conductivity of the as−prepared materials could be improved by directly depositing ultra-thin CuMn_2_O_4_ NSAs−based hierarchical nanostructures on the Ni foam surface. Additionally, this would offer efficient transport routes and rapid ion diffusion channels that would be feasible for electrochemical processes. Therefore, as–prepared binder–free CuMn_2_O_4_ NSAs electrodes with improved electrochemical properties could be a battery-type electrode material for high–rate hybrid supercapacitor applications. Additionally, more advanced experimental research (combining CuMn_2_O_4_ nanostructures with other metal oxides or carbon materials), thorough analyses, and measurement methods are currently being investigated to improve the CuMn_2_O_4_ electroactive material’s properties for electrochemical energy storage applications.

## 4. Conclusions

A facile one-step hydrothermal process was employed to deposit novel CuMn_2_O_4_ NSAs structures on Ni foam. The structural and morphological studies of the as-fabricated CuMn_2_O_4_ NSAs electrode were studied using XRD, SEM, and TEM characterization techniques. The as-developed CuMn_2_O_4_ NSAs electrode displayed a Faradic battery-type redox activity, as evidenced by the potential plateaus from the CV and GCD techniques. As a battery-type electrode material, the CuMn_2_O_4_ NSAs electrode demonstrated exceptional electrochemical capabilities, including the maximum specific capacity (125.56 mA h g^−1^ at 1 A g^−1^), rate capability (84.1% even at 10 A g^−1^), and cycling stability (92.15% over 5000 cycles). Moreover, the as-prepared CuMn_2_O_4_ NSAs electrode material delivered good mechanical stability and flexibility at various bending angles (flat, 45°, and 90°). The as-prepared electrode’s remarkable energy storage performance was due to the hierarchical interconnected nanosheet array architectures, which supplied numerous electroactive sites to facilitate the rapid Faradaic redox reactions. Hence, the excellent electrochemical properties of the CuMn_2_O_4_ NSAs electrode have significant potential for use in high-rate supercapacitors as a battery-type electrode material.

## Data Availability

In this investigation, no fresh data were collected or examined. Sharing of data is not relevant to this subject.
